# The Effects of Biomechanical Loading on the Tibial Insert After Primary Total Knee Arthroplasty: A Systematic Review

**DOI:** 10.3390/jcm14041043

**Published:** 2025-02-07

**Authors:** Alexandru Florin Diconi, Mihai Dan Roman, Adrian Nicolae Cristian, Adrian Gheorghe Boicean, Cosmin Ioan Mohor, Nicolas Catalin Ionut Ion, Bogdan Axente Bocea, Cosmin Adrian Teodoru, George-Calin Oprinca, Sorin Radu Fleaca

**Affiliations:** Faculty of Medicine, Lucian Blaga University of Sibiu, 2A Lucian Blaga Str., 550169 Sibiu, Romania; alexandru.diconi@ulbsibiu.ro (A.F.D.); adrian.cristian@ulbsibiu.ro (A.N.C.); adrian.boicean@ulbsibiu.ro (A.G.B.); cosmin.mohor@ulbsibiu.ro (C.I.M.); nicolascatalinionut.ion@ulbsibiu.ro (N.C.I.I.); bogdanaxente.bocea@ulbsibiu.ro (B.A.B.); adrian.teodoru@ulbsibiu.ro (C.A.T.); georgecalin.oprinca@ulbsibiu.ro (G.-C.O.); radu.fleaca@ulbsibiu.ro (S.R.F.)

**Keywords:** total knee arthroplasty, tibial insert, biomechanical loading, malalignment, posterior tibial slope

## Abstract

**Background/Objectives:** Total knee arthroplasty (TKA) is the gold-standard treatment for advanced knee arthritis, offering pain relief and improved joint function. However, tibial component malalignment, malrotation, and improper biomechanical loading remain critical factors contributing to implant failure, instability, and revision surgeries. This review systematically examines the impact of biomechanical loading on the tibial insert following primary TKA, with a focus on alignment, posterior tibial slope (PTS), and load distribution. **Methods:** A systematic literature search was conducted across the PubMed, Google Scholar, and Web of Science databases following the PRISMA guidelines. Studies investigating the effects of tibial component alignment, varus/valgus deviations, PTS, and load distribution on tibial inserts post-TKA were included. Seven studies meeting the inclusion criteria were analyzed and described narratively. **Results:** The reviewed studies highlighted that varus and valgus malalignment significantly alter tibiofemoral contact pressures and ligament strains, increasing the risk of aseptic loosening and implant failure. Excessive PTS was associated with posterior femoral translation, altered ligament tension, and increased contact stresses on polyethylene (PE) inserts. Kinematically aligned TKA demonstrated reduced tibial force imbalances and improved functional outcomes compared to mechanically aligned TKA. Computational and cadaveric studies revealed that even minor malalignments (e.g., 3° varus or valgus) can cause significant biomechanical changes. **Conclusions:** Biomechanical loading on tibial inserts after primary TKA is highly sensitive to the alignment and PTS. Optimal alignment and controlled biomechanical forces are essential. Kinematically aligned TKA has shown promising effects, preventing aseptic loosening and ensuring long-term implant survival. Further in vivo studies are needed to validate these findings and optimize surgical techniques.

## 1. Introduction

The most effective treatment for advanced knee arthritis is total knee arthroplasty (TKA) [1,2]. Kinematically aligned TKA has been reported to show biomechanical advantages. However, recent evidence [3] suggests that these advantages in functional outcomes compared to mechanical alignment do not reach clinical significance. The number of total knee arthroplasty (TKA) procedures has grown substantially over the past decade and is anticipated to keep increasing. By 2030, some estimates predict that the annual number of procedures in the United States will exceed 3 million. As the number of primary TKAs rises [4], so does the rate of TKA failures, leading to more revision procedures. Studies have explored pre- and postoperative factors, along with implant design, to identify causes of TKA failure [5,6]. It is commonly known that infection, instability, aseptic loosening, polyethylene (PE) wear, stiffness, and peri-prosthetic fracture are the main reasons for TKA revision [1,7,8]. Several other patient-dependent factors, such as gender, age, body mass index (BMI) [9], socio-economic factors, associated comorbidities, correct positioning of tibial and femoral components, correct alignment of the implant, and design influence the outcome of TKA [10]. However, the most frequent cause of poor results following TKA is the incorrect alignment of tibial and femoral components, causing patients to experience stiffness, pain, instability of the tibiofemoral (TF) joint, and aseptic loosening and ultimately leading to the necessity of revision [10]. Consequently, different tibial PE insert designs have been developed to enhance natural knee biomechanics, ensuring optimal biomechanical loading after primary TKA and extending the longevity of the prosthesis [11]. The two most common approaches for TKA are classic posterior stabilized (PS) and cruciate retaining (CR) tibial inserts [12], which usually have positive results, while the material’s aging and use can have unfavorable effects [13]. Cemented TKA has traditionally been the gold standard, providing reliable joint stability and functional restoration. However, in younger, more active, and obese populations, the durability of cemented fixation may be suboptimal. Advancements in surgical techniques and the development of cementless prostheses present compelling alternatives [14]. However, in a recent narrative review by Wilczynski et al. [15], 14 studies published between 2010 and 2023 were analyzed, encompassing a total of 783 knees: 356 with cemented fixation and 399 with cementless fixation. The average follow-up period across these studies was 8.4 years, ranging from 2.0 to 16.6 years. The review found no significant difference in revision rates between cemented and cementless fixation methods. The patient-reported functional scores, including measures of pain and knee function, were similar for both fixation methods, and both methods were associated with similar complication rates.

Biomechanical loading refers to the physical forces that are exerted on the body across various levels, including systems, organs, tissues, as well as molecular and cellular structures. The variables influencing mechanical loading can be defined by the magnitude, duration, frequency, type, and direction of force application [16]. With three translational and three rotational degrees of freedom, the knee is a modified hinge joint [17]. Different activities cause the medial and lateral condyles to translate to different degrees across knee range of motion. Fluoroscopic studies indicate that during normal gait, the lateral condyle shifts −11.8 mm, while the medial condyle moves −4.7 mm. In deep bending, the lateral condyle travels −16.3 mm, and the medial condyle moves −4.5 mm. Consequently, the femur and tibia experience posterior translation and internal rotation relative to the distal femur. Under normal conditions, the knee can flex between 140 and 160 degrees [18,19]. “Femoral roll-back” refers to the difference in movement between the medial and lateral condyles during knee flexion. The motion is primarily rotational in the first 15 to 20° but then changes to posterior translation of the femur beyond that point. This is opposite to what is called the “screw home mechanism”, in which the femur translates anteriorly and the tibia rotates externally [20]. However, the main debate is whether or not the posterior cruciate ligament (PCL) needs to be sacrificed in TKA [21]. The primary role of the PCL in the knee is to prevent excessive posterior translation of the tibia. Additionally, it helps limit excessive tibial internal rotation between 90° and 120° of flexion. An intact and functional PCL prevents excessive posterior translation of the tibia relative to the femur and allows for acceptable amounts of femoral rollback, according to cadaveric research [22]. This provides credence to the idea that after TKA, maintaining the PCL enables more natural knee kinematics, particularly enabling the screw home process to take place in the last 20 degrees of extension [23,24].

The knee experiences significant biomechanical forces during daily activities, varying with movements like walking, running, or climbing. Factors such as anatomical abnormalities (e.g., stiff hip, tibial varus deformity), posture, body weight, lifestyle, and movement speed also influence these forces. During routine activities, compressive forces on the knee can reach 6–7 times an individual’s body weight [25]. During walking, peak biomechanical forces in the knee occur just after the heel strike and during push-off. After push-off, the compressive forces drop to nearly zero as the swing phase begins. During push-off, the compressive, shear, and rotational forces peak, with the anterior shear forces equaling the body weight after heel strike and posterior shear forces being double that of the body weight during push-off. Medial shear forces transition laterally, reaching a peak of 1.5 times the body weight, while the rotational torque exerts external rotation forces that attempt to dislocate the knee. These peak forces occur with the knee flexed between 0 and 20°, aligning with the tibial articular surface’s natural 7–10° flexion. This alignment plays a crucial role in the design and placement of tibial components in knee replacements. Furthermore, at peak loading, the knee is almost fully extended, with ligaments being under maximum tension [26,27].

For rotational alignment of tibial component (tibial insert and plateaus), a range of anatomical landmarks can be used, including the medial border and medial third of the tibial tuberosity, the posterior tibial condylar line, the anterior tibial crest, the second ray, and the first web space of the foot [28]. Aligning the tibial component with the tibial tubercle is a common method based on anatomical landmarks. However, a limitation of this approach is that it does not account for femoro-tibial kinematics. To address this, the range of motion (ROM) technique was introduced, where the rotational alignment of the tibial tray is adjusted to ensure that it conforms to the femoral component during repeated flexion–extension cycles [29]. The primary goal of tibial component rotational alignment in TKA is to ensure parallelism on the coronal plane between the femoral transepicondylar axis (TEA) and the mediolateral (ML) axis of the tibial component, avoiding internal or external rotational errors. However, achieving this ideal alignment throughout the ROM is challenging because the TEA, whether surgical or anatomical, behaves cylindrically, and the tibial plateau undergoes significant internal rotation during regular daily activities [30].

During TKA, the surgeon removes the knee’s intrinsic stability, provided by the geometry of the articular surfaces and menisci, to create enough space for the prosthesis. Additionally, some extrinsic stability is often compromised during the procedure, when the cruciate ligaments are sacrificed, as is its function of providing stabilization for the knee muscles [31]. Biomechanically, maintaining natural, anatomical motion after total knee arthroplasty (TKA) helps prevent the mechanical loosening of prosthetic components. When motion is not excessively restricted, rotational and shear forces are absorbed by the soft tissues rather than the prosthesis, which reduces stress on the component fixation and lowers the risk of loosening [26,27]. However, this stability is mainly affected by biomechanical loading on the tibial component after primary TKA. The main factors that can induce biomechanical loading on the tibial component after primary TKA are the rotational positioning of the tibial component, balancing the knee in varus/valgus deviations, and the increase in the posterior slope [30,32,33]. Therefore, the purpose of this study was to review the studies that have been carried out on the effect of biomechanical loading on tibial insert following TKA.

## 2. Methods

To identify relevant studies on the evaluated topic, the PRISMA guidelines for systematic reviews were adhered to [34]. A systematic literature search was conducted across PubMed (including MEDLINE), Google scholar, and Web of Science (WoS) up to 31 November 2024. A literature review was performed by three authors using the following key words: “primary total knee arthroplasty” or “primary TKA”, “biomechanical loading”, “tibial insertion”, “tibial insert“, “tibial component”, “malposition”, “malrotation”, “positioning”, “varus”, “valgus”, and “posterior slope”.

### Inclusion and Exclusion Criteria

**Figure 1:**
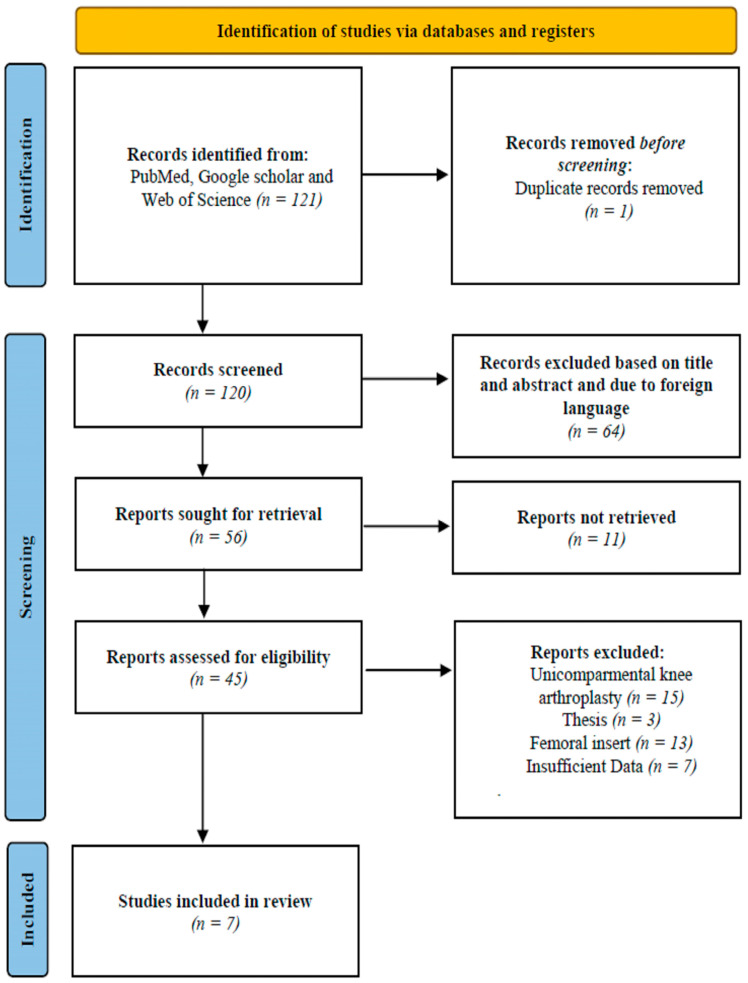
A PRISMA flow chart of the study selection process and inclusion and exclusion criteria.

Articles that were considered for inclusion had to meet the following criteria: they must be peer-reviewed, be written in English, present studies that refer only to primary TKA, and be research papers about tibial component positioning. Additionally, the studies needed to have either a cross-sectional design or use an intervention. Studies about unicompartmental knee arthroplasty (UKA), meta-analyses or systematic reviews, theses, studies that refer to an insertion of the femoral component, and studies not written in the English language were excluded (Figure 1).

## 3. Results and Discussion

There were 121 studies identified from the initial search using the PubMed, Google scholar, and WoS databases. No other records were identified through other sources, and only one duplicate article was removed. The authors screened the remaining 120 titles and abstracts and excluded 64 articles that were not relevant to the topic and research question or were written in a foreign language. Only 45 articles remained eligible, and the authors reviewed the full-text articles for inclusion. Following this, seven articles remained and were subsequently included in this study. The results of this process can be seen in Figure 1. We attempted to retrieve the remaining 56 articles, and 45 were assessed for eligibility.

The findings from all the included studies are summarized in Table 1 and further explained and discussed in the following narrative review. Unless stated otherwise (e.g., no observed change), the results presented in Table 1 were considered statistically significant (*p* < 0.05).

Kinematic parameters and contact pressures play a crucial role in knee function following TKA. More natural knee kinematics, such as femoral external rotation and posterior translation during flexion, are associated with improved functional outcomes and higher patient satisfaction after TKA [40]. Alterations in load-bearing patterns can affect the contact area and pressures, which are associated with aseptic loosening—the primary cause of surgical failure and the need for prosthesis revision [41,42]. Stress shielding, resulting from an altered load distribution, may trigger bone remodeling, a key factor contributing to prosthesis loosening [43]. An excessive contact pressure can also accelerate PE prosthesis wear, producing debris and wear particles. This debris can trigger a biological response, resulting in bone resorption and osteolysis, both of which are major factors in prosthesis loosening [44].

Malalignment/malposition or malrotation was the seventh most common reason for revision in a retrospective review of 820 revision TKA procedures [45]. Furthermore, malalignment can impact joint loading, leading to ligament instability and prosthesis loosening. Coronal malalignment of the tibial component in TKA may also necessitate additional revision surgery [46]. Approximately 28% of individuals have coronal malalignment, even when the procedure is performed by skilled surgeons [47]. Even with advancements in surgical instruments, procedures, and implant designs, component malpositioning remains a direct cause of revisions [47].

Even with deviations below 3° or under the ideal alignment, wear acceleration has been observed, highlighting the complexity of wear mechanisms in TKA. While achieving precise alignment remains a critical surgical goal, other factors may contribute significantly to wear, such as patient-specific biomechanics, the implant’s material properties, activity levels, and localized loading conditions. For instance, Srivastava et al. [48] found that varus malalignment below 3° resulted in accelerated wear, even in cases where the overall alignment was nearly ideal. Similarly, Berend et al. [49] demonstrated that varus alignment above 3° leads to medial bone collapse, emphasizing the sensitivity of PE inserts to minor alignment deviations.

Additionally, research has indicated that even well-aligned components can generate polyethylene wear debris due to high localized stresses [43]. These localized stresses may arise from uneven load distributions, soft tissue imbalances, or variations in patient gait mechanics. A study by Fang et al. [50] further supports this observation, linking a varus/valgus deviation above 3° from the mechanical axis to abnormal stress, prosthesis deterioration, and aseptic loosening. Furthermore, D’Lima et al. [46] demonstrated that small rotational malalignments could result in uneven wear patterns and increased ligamentous strain, further contributing to wear and instability.

The interplay between alignment and other biomechanical factors underscores the need for a comprehensive approach to reducing wear in TKA. While surgical precision in achieving alignment is paramount, patient-specific factors and implant design considerations must also be addressed to optimize long-term prosthesis survival and functional outcomes.

In a musculoskeletal (MSC) study by Chen et al. [32], it was found that the peak total TF contact force at the maximum load bearing increased by 11.0% with a 5° varus alignment of the tibial insert. The PTS of the tibial insert only affected the total TF contact force during the swing phase of the gait cycle. The total TF contact force at peak knee flexion increased by 14.7% with 5° anterior tilting of the tibial insert and decreased by 12.6% with 5° posterior tilting. During the first half of the stance phase, the force change was 7.9% with 5° anterior tilting of the tibial insert. However, the total TF contact forces were not significantly affected by variations in the internal/external malrotation of the tibial component. Furthermore, the peak medial contact force increased by 36.2% with a 5° varus alignment of the tibial insert, while the lateral contact force at maximum load bearing decreased by 68.0% with the same alignment [32]. These results are in line with studies by Werner et al. [35] and Berend et al. [49], which suggest that a 3° or 5° varus/valgus rotation of the tibial insert significantly altered the TF medial–lateral loading distribution. In a recent study by Roth et al. [36], it was shown that kinematically aligned TKA reduced the average total tibial forces to 116 N from 0° to 120° of flexion and limited the average difference in tibial forces between compartments to 29 N across the same range of motion. These results, particularly the low average total tibial forces, indicate that aligned TKA reduces the risk of excessive biomechanical loading that could damage the prosthesis and soft tissue tightness, which may cause ongoing pain, stiffness, and limited ROM. In all thirteen kinematically aligned TKAs, the contact points in both compartments shifted posteriorly by an average of 14 mm in the medial compartment and 18 mm in the lateral compartment as the knee moved from 0° to 120° of flexion (*p* < 0.0001) [36]. Although in vivo confirmation of this study is needed, these findings suggest that kinematically aligned TKA may better minimize tibial force differences between compartments compared to mechanically aligned TKA. This could explain why kinematically aligned TKA is associated with higher patient satisfaction and function, as well as why patients are three times more likely to report that their knee feels normal compared to those with mechanically aligned TKA (Table 1).

Research indicates that increasing the PTS enhances the posterior femoral translation, flexion gap, and maximum flexion angle in CR TKA [51,52,53]. While finite element models (FEMs) predict knee contact pressures at varying PTSs, there is limited in vitro evidence of its effect in PS TKA [42]. Cadaveric studies on PTS in PS-TKA are scarce, and the benefits of increasing the PTS in this context remain debated [54,55,56]. A retrospective study was performed by Wang et al. [33] on nine human cadaveric knee specimens that underwent PS-TKA with the PTS set at 3°, 6°, and 9°. The TF kinematics and contact pressures were assessed during knee flexion from 0° to 120° in 10° increments, with an axial load of 1000 N being applied at each angle. After TKA, the TF contact area reduced from 586.2 mm^2^ to 130.2 mm^2^, while the average and peak contact pressures rose from 1.85 MPa and 5.39 MPa to 7.56 MPa and 17.98 MPa, respectively. A larger PTS was associated with a greater contact area and lower mean and peak contact pressures. The femoral rotation differences before and after TKA exceeded 9.9°, and the posterior translation of the lateral condyle was greater with a larger PTS. Overall, the root mean square (RMS) differences in posterior translation before and after TKA were more than 11.4 mm [42]. These results indicate that TKA with a larger PTS leads to greater posterior femoral translation, a larger contact area, and lower contact pressure, which could lead to positive biomechanical effects on the knee joint. This suggests that, when performed carefully, increasing the PTS could be beneficial for PS TKA. However, another study by Kang et al. [37], in which a validated TKA computational model was used, showed that an excessively large PTS should be avoided, as it can lead to progressive loosening of the knee joint due to a decrease in collateral ligament tension and failure of the posterior region in a PE insert. Authors have shown that as the PTS increased, the maximum force on the quadriceps and patellofemoral (PF) contact stress decreased. The TF joint biomechanics shifted more posteriorly, with the medial and lateral contact points on the PE insert moving to posterior regions. A higher PTS also reduced the force on the collateral ligaments while increasing the contact stress on the posterior of the PE insert. Furthermore, a study by Bai et al. [57] showed that antero-posterior (A-P) instability may occur, causing posterior subluxation of the tibial component. This increases the shear stresses on the posterior part of the PE tibial insert, potentially compromising the implant’s performance (Table 1).

Restoring the mechanical axis in TKA is essential for proper weight distribution and long-term functionality. Traditionally, this axis extends from the femoral head to the center of the ankle. However, factors such as sagittal balancing during surgery and hindfoot malalignment can introduce valgus stress, affecting the overall loading axis of the lower limb [58,59]. Valgus forces impact the medial collateral ligament, the primary restraint against valgus angulation, which can weaken over time due to repeated stress [38]. Bryant et al. [38] examined the effects of the biomechanical load on the valgus on eight cadaveric knees after TKA. The knees were tested at 0°, 30°, and 60° of flexion under both neutral alignment and 5° valgus. Contact areas and pressures were measured using Fuji pressure-sensitive film, while the strain on the medial collateral ligament (MCL) was evaluated with a Microscribe digitizing system. Valgus loading significantly increased lateral TF pressures (*p* < 0.05) across all flexion angles, while the patellofemoral contact characteristics showed no significant changes (*p* > 0.05). The strain along the anterior and posterior borders of the MCL significantly increased at all flexion angles. These results suggest that valgus loading increases the joint contact pressure and MCL strain with greater knee flexion, potentially contributing to implant instability. Significant increases in strain were observed in the anterior portion of the MCL between neutral and valgus conditions at 0° (2.5 ± 0.6%; *p* = 0.006), 30° (3.1 ± 0.6%; *p* = 0.001), and 60° (3.7 ± 0.8%; *p* = 0.005) of knee flexion. A 5° valgus angle led to higher lateral TF contact pressure and increased MCL strain, raising the risk of instability and potential failure in TKA [38]. By comparison, an FEM study by Suh et al. [39] also found an effect of not only valgus, but also varus, on the biomechanical loading of tibial inserts. The authors examined the maximum contact stress on tibial PE inserts in neutral and malaligned models during the stance phase of the gait cycle. In varus alignment, the peak medial contact stress increased by 24.0% at 3° and 35.0% at 5°, while the lateral contact stress decreased by 17.4% and 27.3%, respectively. Conversely, in valgus alignment, the trend was reversed, with the medial contact stress decreasing by 37.2% at 3° and 50.7% at 5°, while the lateral contact stress increased by 13.3% and 16.9%, respectively. The highest stress on the PE insert occurred on the medial side in cases of varus malalignment. In valgus malalignment, increased force on the medial ligament indicated a higher risk of total TKA failure due to ligament failure [39]. This is confirmed by the results of other studies, in which it was proven that varus and valgus malalignments result in high wear in the medial compartment and failure due to ligament instability [50] (Table 1).

However, it is known that performance measures and gait analysis are commonly used to evaluate postoperative knee function, but they focus on overall motor function rather than specific movements, contact pressures, and biomechanical loading within the knee joint [60]. While FEM simulations have been used to estimate knee kinematics and contact pressures, their validity has been questioned [42,61]. In vivo measurement of the contact pressure in the knee is not feasible due to practical or ethical concerns, so in vitro biomechanical experiments are often used instead. These experiments have successfully measured knee biomechanics and contact pressures in cadaveric studies [51,62,63]. The advantage of in vitro experiments lies in their ability to test various surgical procedures, such as osteotomy, at different angles, which is challenging to achieve in vivo, and most of the studies that we analyzed in our review were cadaveric studies, whose results complement in vivo ones the best.

## 4. Conclusions

Optimizing kinematic parameters and contact pressures in TKA is essential to improving functional outcomes, reducing prosthesis wear, and preventing aseptic loosening, the leading cause of surgical failure and revision.

Malalignment of the tibial component in TKA significantly impacts joint loading, ligament stability, and prosthesis longevity, contributing to aseptic loosening, accelerated wear, and revision surgeries. Even minor deviations, such as a 3° varus alignment, can cause an abnormal stress distribution, medial bone collapse, and increased contact forces. Kinematically aligned TKA has shown promising results in reducing tibial force imbalances and lowering overall tibial forces. However, while TKA demonstrates several improved functional outcomes, these improvements do not reach clinical significance when compared to mechanically aligned TKA. As such, conclusions about the superiority of KA in terms of functional outcomes should be drawn with caution.

Increasing the PTS in PS TKA enhances the posterior femoral translation, flexion gap, and maximum flexion angle while reducing the mean and peak contact pressures and increasing the contact area. However, an excessively large PTS can lead to complications such as reduced collateral ligament tension, increased postcontact stress, and anterior–posterior instability, potentially compromising the implant’s performance and longevity. Careful optimization of the PTS is crucial to achieve improved biomechanics and prevent adverse outcomes.

Restoring the mechanical axis in TKA is essential for a balanced weight distribution and long-term implant stability. Valgus and varus malalignments disrupt TF contact pressures and lead to strain on the MCL, increasing the risk of implant instability and failure. Valgus alignment elevates the lateral TF pressures and MCL strain, while varus alignment amplifies the medial contact stress and ligament tension. Both malalignments contribute to uneven wear patterns, ligament instability, and higher failure rates, emphasizing the importance of precise alignment for optimal TKA outcomes.

## Figures and Tables

**Table 1:** Description of included studies.

Authors	Year	Study Design	Population	Type of Intervention	Results	Effect on Biomechanics
Chen et al. [32]	2015	Musculoskeletal study	Human female knees	Effect of tibial insert malrotation on biomechanical loading	Peak TF contact force increased by 11.0% with a 5° varus alignment of the tibial insert.	Greater than 3° varus malrotation of the tibial component may lead to medial bone collapse
Werner et al. [35]	2005	Retrospective cadaveric study	Human cadaveric knees (*n* = 7)	The effect of valgus/varus malalignment on load distribution in TKA	A 3° variation in angulation caused biomechanical changes in the medial and lateral compartments of the tibial component.	Tibial contact pressures measured during a trial reduction could predict the contact mechanics under higher loading conditions
Roth et al. [36]	2017	Retrospective cadaveric study	Human cadaveric knees (*n* = 13)	Effect of tibial forces on aligned TKA	Contact locations shifted posteriorly by an average of 14 mm in the medial compartment and 18 mm in the lateral compartment from 0° to 120° of flexion.	Alignment methods reduce high tibial forces, minimize force differences between compartments, and limit anterior tibial contact shift during passive flexion
Wang et al. [33]	2020	Retrospective cadaveric study	Human cadaveric knees (*n* = 9)	Different PTSs were compared	After TKA, the TF contact area decreased from 586.2 mm^2^ to 130.2 mm^2^, while contact pressures increased.	TKA with a larger PTS results in more posterior femoral translation, a larger contact area, and less contact pressure
Kang et al. [37]	2017	Computational study	Computational model	Forces on the quadriceps, tibial posterior translation, the PE insert, and knee joint were compared	Increase in the PTS led to decrease in medial and collateral ligaments, as well as decrease in the maximum force on the quadriceps.	Excessive increase in PTS may cause progressive loosening of the knee joint due to reduced collateral ligament tension and failure of the posterior of the PE insert
Bryant et al. [38]	2014	Retrospective cadaveric study	Human cadaveric knees (*n* = 8)	Effect of increased valgus after TKA	Increases in valgus loading led to strain of anterior MCL when compared to neutral conditions at 0° (2.5%), 30° (3.1%), and 60° (3.7%) of knee flexion.	A 5° valgus angle was associated with increased lateral tibiofemoral contact pressures and increased strain on the MCL
Suh et al. [39]	2017	Computational FEM study	Computational model	Effect of varus and valgus malalignment on biomechanics after TKA	In varus alignment, the medial contact stress increased by 24.0% at 3° and 35.0% at 5°, while the lateral stress decreased. In contrast, valgus alignment decreased the medial stress by 37.2% at 3° and 50.7% at 5°, while the lateral stress increased.	Varus malalignment caused maximum stress on the medial PE insert, while valgus malalignment increased the medial ligament force, raising the risk of TKA failure

TKA—total knee arthroplasty; PTS—posterior tibial slope; FEM—finite element model; TF—tibiofemoral.

## Data Availability

Data are contained within the article.

## Abbreviations

The following abbreviations are used in this manuscript:

TKA	Total knee arthroplasty
PTS	Posterior tibial slope
PE	Polyethylene
BMI	Body mass index
TF	Tibiofemoral
PS	Posterior-stabilized
CR	Cruciate-retaining
PCL	Posterior cruciate ligament
ROM	Range of motion
TEA	Transepicondylar axis
ML	Mediolateral
FEM	Finite element model
MSC	Musculoskeletal
A-P	Antero-posterior
RMS	Root mean square
PF	Patellofemoral
